# Molecular Shadows of Per- and Polyfluoroalkyl Substances (PFASs): Unveiling the Impact of Perfluoroalkyl Substances on Ovarian Function, Polycystic Ovarian Syndrome (PCOS), and In Vitro Fertilization (IVF) Outcomes

**DOI:** 10.3390/ijms26146604

**Published:** 2025-07-10

**Authors:** Charalampos Voros, Diamantis Athanasiou, Ioannis Papapanagiotou, Despoina Mavrogianni, Antonia Varthaliti, Kyriakos Bananis, Antonia Athanasiou, Aikaterini Athanasiou, Georgios Papadimas, Athanasios Gkirgkinoudis, Kyriaki Migklis, Dimitrios Vaitsis, Aristotelis-Marios Koulakmanidis, Charalampos Tsimpoukelis, Sofia Ivanidou, Anahit J. Stepanyan, Maria Anastasia Daskalaki, Marianna Theodora, Panagiotis Antsaklis, Dimitrios Loutradi, Georgios Daskalakis

**Affiliations:** 1 Department of Obstetrics and Gynecology, ‘Alexandra’ General Hospital, National and Kapodistrian University of Athens, 80 Vasilissis Sofias Avenue, 11528 Athens, Greece; depy.mavrogianni@yahoo.com (D.M.); antonia.varthaliti@hotmail.com (A.V.); tgkirgki@gmail.com (A.G.); aristoteliskoulak@gmail.com (A.-M.K.); tsimpoukelischa@gmail.com (C.T.); md181341@students.euc.ac.cy (M.A.D.); martheodr@gmail.com (M.T.); panosant@gmail.com (P.A.); gdaskalakis@yahoo.com (G.D.); 2 IVF Athens Reproduction Center V. Athanasiou, 15123 Maroussi, Greece; diamathan16@gmail.com (D.A.); antoathan16@gmail.com (A.A.); diamathan17@gmail.com (A.A.); 3 Athens Medical School, National and Kapodistrian University of Athens, 15772 Athens, Greece; gpapamd@hotmail.com (I.P.); dr.georgepapadimas@gmail.com (G.P.); kyriaki.migklis@gmail.com (K.M.); vaitsisdim@gmail.com (D.V.); info@ivanidou.gr (S.I.); anahitstepanyanj@gmail.com (A.J.S.); loutradi@otenet.gr (D.L.); 4 King’s College Hospital NHS Foundation Trust, London SE5 9RS, UK; kyriakos.bananis@nhs.net; 5 Fertility Institute-Assisted Reproduction Unit, Paster 15, 11528 Athens, Greece

**Keywords:** perfluoroalkyl substances, female infertility, ovarian function, polycystic ovary syndrome, in vitro fertilization, endocrine disruptors, epigenetic effects

## Abstract

Per- and polyfluoroalkyl substances (PFASs) comprise a diverse array of synthetic chemicals that resist environmental degradation. They are increasingly recognised as endocrine-disrupting compounds (EDCs). These chemicals, found in non-stick cookware, food packaging, and industrial waste, accumulate in human tissues and fluids, raising substantial concerns regarding their impact on female reproductive health. Epidemiological studies have demonstrated associations between PFAS exposure and reduced fertility; nevertheless, the underlying molecular pathways remain inadequately understood. This narrative review investigates the multifaceted effects of PFASs on ovarian physiology, including its disruption of the hypothalamic–pituitary–ovarian (HPO) axis, alteration of anti-Müllerian hormone (AMH) levels, folliculogenesis, and gonadotropin receptor signalling. Significant attention is directed towards the emerging association between PFASs and polycystic ovarian syndrome (PCOS), wherein PFAS-induced hormonal disruption may exacerbate metabolic issues and elevated androgen levels. Furthermore, we analyse the current data regarding PFAS exposure in women undergoing treatment based on assisted reproductive technologies (ARTs), specifically in vitro fertilisation (IVF), highlighting possible associations with diminished oocyte quality, suboptimal embryo development, and implantation failure. We examine potential epigenetic and transgenerational alterations that may influence women’s reproductive capabilities over time. This study underscores the urgent need for further research and regulatory actions to tackle PFAS-related reproductive toxicity, particularly in vulnerable populations, such as women of reproductive age and those receiving fertility treatments.

## 1. Introduction

Per- and polyfluoroalkyl substances (PFASs) constitute a substantial category of synthetic organofluorine chemicals. Among these are prominent legacy compounds, like perfluorooctanoic acid (PFOA) and perfluorooctane sulfonate (PFOS) [1]. Other recent short-chain options include hexafluoropropylene oxide dimer acid (HFPO-DA), commonly referred to as GenX. Short- and long-chain PFASs are employed in industry, although they exhibit significant differences in their accumulation in organisms, persistence, and toxicity. Long-chain PFASs, such as PFOA and PFOS, consist of eight or more carbon atoms. They are recognised for their significant bioaccumulation potential and extended half-lives in human tissues. Epidemiological and experimental studies have demonstrated a more consistent association between these substances and adverse reproductive outcomes. Conversely, short-chain PFASs exhibit greater water solubility and typically exit the body more rapidly. Nevertheless, recent data indicates that certain short-chain alternatives may still disrupt the endocrine system, oxidative equilibrium, and ovarian signalling pathways, despite their shorter duration of action. It is crucial to acknowledge that we do not yet fully comprehend their toxicological profiles, necessitating additional research, particularly on female fertility. PFASs are referred to as “forever chemicals” because of their exceptional chemical stability and resistance to enzymatic degradation [2]. They can persist in the environment and within human tissues for years. Due to their extensive application in industry, consumer products, and firefighting foams, they are now ubiquitous in the environment and present in human biological matrices such as blood, urine, breast milk, and follicular fluid [3].

Recent scientific investigation has concentrated on the capacity of PFASs to act as endocrine-disrupting chemicals (EDCs), interfering with hormonal signalling at many levels of the hypothalamic–pituitary–gonadal (HPG) axis [4]. These substances can alter the central regulation of reproduction by disrupting the secretion of kisspeptin and gonadotropin-releasing hormone (GnRH) in the hypothalamus, potentially obstructing the pulsatile release of luteinizing hormone (LH) and follicle-stimulating hormone (FSH) from the anterior pituitary [5]. These alterations may result in delayed puberty, irregular menstruation, and cessation of ovulation. PFASs have been demonstrated to exert detrimental local effects on the ovary by disrupting the function of steroidogenic enzymes, including steroidogenic acute regulatory protein (StAR), 3β-hydroxysteroid dehydrogenase (HSD3B1), and aromatase (CYP19A1) [6]. Experimental models indicate that PFASs may diminish the expression of CYP19A1, reduce oestradiol synthesis, and impair the functionality of granulosa and theca cells. This may impair folliculogenesis and the developmental capacity of oocytes [7].

Extensive study indicates that PFASs function at the molecular level by activating the nuclear receptor peroxisome proliferator-activated receptor gamma (PPARγ) [8]. In granulosa cells, the activation of PPARγ has been shown to inhibit FSH-dependent cAMP-protein kinase A signalling, leading to reduced oestradiol synthesis and the suppression of preovulatory follicle maturation [9]. Furthermore, PFASs have been shown to alter the expression and function of gap junction proteins, such as connexin 43 (Cx43), thereby impairing intercellular communication between oocytes and cumulus cells. This disruption may negatively impact the transfer of ions, growth hormones, and energy substrates vital for oocyte maturation and developmental competence [10].

Anti-Müllerian hormone (AMH), a key marker of ovarian reserve produced by small developing follicles, appears to be influenced by PFASs. Research indicates that women exposed to specific drugs exhibit reduced levels of AMH, suggesting impaired follicular recruitment [11]. Additional research indicates that elevated levels of AMH may inhibit follicular development, particularly in women diagnosed with polycystic ovarian syndrome (PCOS). Moreover, PFASs may reduce the expression of the FSH receptor (FSHR) or desensitise its signalling cascade, further impeding follicle growth and response to gonadotropin stimulation [12,13].

Women with PCOS may have heightened sensitivity to PFAS exposure. This syndrome, characterised by hyperandrogenism, oligo/anovulation, and insulin resistance, may be aggravated by the endocrine and metabolic disruptions induced by PFASs [14]. Recent in silico toxicogenomic studies have shown that PFASs interact with a broad array of genes associated with steroidogenesis and cell cycle regulation, including CCNB1 and SRD5A1, suggesting a potential mechanistic link between PFAS exposure and the pathophysiology of PCOS [15]. Furthermore, specific branched-chain and short-chain PFAS isomers have been associated with increased risks of PCOS, especially in overweight and obese women, suggesting potential gene–environment and metabolic interactions [16].

PFASs pose a concern to women employing assisted reproductive technologies (ARTs), particularly in vitro fertilisation (IVF). Clinical trials have identified PFASs in the follicular fluid of IVF patients, indicating that increased levels correlate with decreased oocyte yield, a smaller number of two-pronuclear zygotes, poorer embryo cleavage, and lowered clinical pregnancy rates [17]. These observations indicate the possible involvement of PFAS in diminishing the effectiveness of ARTs, likely via modifying gonadotropin receptor signalling, oocyte–cumulus communication, and hormone synthesis during the peri-ovulatory phase [18].

Besides causing acute ovarian harm, PFASs may result in lasting and transgenerational effects through epigenetic mechanisms. Alterations in DNA methylation patterns, histone alterations, and the unregulated synthesis of microRNAs (miRNAs) have been seen in both animal and in vitro models following exposure to PFASs [19,20]. These modifications may affect not only the current reproductive ability of exposed individuals but also the reproductive health of their offspring. Moreover, the placental transfer of PFASs and their interference with foetal hormonal programming intensify apprehensions about the developmental origins of health and disease [21].

Despite increasing epidemiological and experimental evidence, the molecular targets of PFASs in the female reproductive system remain incompletely understood. While most studies focus on long-chain PFASs, the effects of recently discovered short-chain alternatives are insufficiently defined. Furthermore, there is a paucity of systematic research examining the cumulative effects of PFAS combinations, which more accurately reflect real human exposure. This narrative review aims to synthesise existing research about the molecular impacts of PFASs on ovarian function, the exacerbation of PCOS characteristics, and their repercussions on ART outcomes, namely IVF. This review seeks to identify critical knowledge gaps and outline potential research directions in female reproductive toxicology, focussing on hormonal regulation, folliculogenesis, oocyte quality, and epigenetic disruption.

## 2. PFAS as Endocrine Disruptors: Mechanisms of Action

PFASs are becoming recognised as endocrine-disrupting chemicals (EDCs) that can alter hormonal equilibrium and adversely affect women’s reproductive health through complex molecular mechanisms. These chemicals interfere with signalling at multiple levels of the HPG axis and directly affect ovarian tissue, specifically granulosa cells and theca cells [22,23]. PFASs may affect the central axis by modifying the neural circuits that govern reproductive neuroendocrine control. PFASs selectively influence the transcriptional regulation of KISS1 and KISS1R, altering the excitation of GnRH neurones. Studies have demonstrated that PFAS exposure reduces kisspeptin expression in the arcuate nucleus (ARC) and anteroventral periventricular (AVPV) regions of the hypothalamus, thus decreasing GnRH pulsatility [7]. The inhibition of GnRH release diminishes the subsequent activation of gonadotrophs in the anterior pituitary, leading to impaired synthesis and secretion of LH and FSH. PFASs may induce inflammation in the hypothalamus by activating the TLR4 and NF-κB signalling pathways. This may result in neuroimmune-mediated suppression of GnRH neuron activity [24].

In animal models, exposure to PFOS and PFOA diminishes the amplitude and frequency of the LH surge, partially mediated by the modified expression of Gnrh1 and its receptor Gnrhr in the pituitary gland. Reduced amounts of LH are associated with insufficient activation of the LH receptor in TCs. This inhibits androgen synthesis via CYP17A1, thereby impacting oestrogen production in granulosa cells. Additionally, PFASs have been associated with reduced E2 levels due to the inhibition of aromatase activity and the impaired responsiveness of granulosa cells to FSH [25]. These hormonal changes correlate with alterations in the expression of circadian clock genes (e.g., Per2, Clock), perhaps further disrupting the temporal regulation of GnRH/LH release. Delays in vaginal opening and modified cyclicity due to PFASs have been linked to changes in the expression of oestrogen receptor alpha and androgen receptors in the hypothalamus. This indicates that feedback dysregulation at the central level is attributable to nuclear receptors [26].

PFASs directly affect granulosa cells and theca cells in the ovary by disrupting the signalling pathways regulated by follicle-stimulating hormone receptors and luteinizing hormone receptors. FSH binding to the FSHR typically activates the cAMP/PKA pathway, resulting in elevated levels of CYP19A1, StAR, and HSD3B1 [6,7].

Exposure to PFASs diminishes the cAMP production induced by FSH and disrupts CREB phosphorylation, leading to a decrease in the transcription of enzymes responsible for E2 synthesis [27]. In testicular cells, decreased LH-LHR interaction diminishes androgen production by suppressing CYP17A1 and CYP11A1 [28]. This complicates the procurement of substrates necessary for GCs to convert into E2. PFASs may reduce membrane fluidity and impair cholesterol transport, thus hindering mitochondria from assimilating the precursors essential for steroidogenesis [29]. PFASs may significantly impede signal transmission by disrupting lipid rafts and G-protein coupling inside the GC membrane environment. The cumulative impact is a diminished steroidogenic response, hindering follicular growth and the selection of the optimal follicle [30,31].

PFASs stimulate PPARγ, resulting in decreased aromatase expression and inhibition of GC differentiation. Via generating a PPARγ–RXR heterodimer, PFASs can inhibit the transcription of the FSHR and impede the growth of GCs by altering the amounts of cyclins (such as CCND2) and CDKs. Exposure to PFASs alters the equilibrium between proteins that promote cellular survival (such as BCL2) and proteins that facilitate cellular apoptosis (such as BAX and CASP3) [32]. This results in increased GC apoptosis and follicular atresia. Besides influencing the PI3K/AKT and MAPK/ERK pathways, PPARγ activation inhibits the phosphorylation of FOXO3a and translocates it to the nucleus, thereby preventing follicular activation and progression [33]. These molecular changes resemble the phenotypes of early ovarian insufficiency and may clarify the observed reductions in AMH levels and the antral follicle count in affected women.

The gap junction intercellular communication (GJIC) between oocytes and cumulus cells, mediated by Cx43, is impaired, resulting in reduced diffusion of cAMP, ATP, and short RNAs that are crucial for coordinating nuclear and cytoplasmic maturation [34,35]. Compromised gap junction integrity impairs GDF9 and BMP15 signalling, which are crucial oocyte-derived factors regulating cumulus cell growth and metabolic support. PFASs reduce the expression of Gja1, the gene encoding Cx43, as well as essential transcriptional co-factors such as FOXL2, thereby further destabilising bidirectional communication [33,36]. Thus, impaired cumulus–oocyte signalling delays germinal vesicle breakdown, obstructs MPF activation, and leads to meiotic incompetence. This functional distinction may clarify the reduced fertilisation and blastocyst formation rates observed in IVF patients with increased PFAS exposure [37].

Disruption of AMH expression intensifies follicular dysregulation. PFASs may alter AMHR2 signalling in preantral follicles, potentially impacting SMAD-dependent transcription of genes essential for folliculogenesis. Unregulated AMH inhibits aromatase production and leads to the accumulation of tiny antral follicles, indicative of PCOS [38]. Furthermore, elevated AMH levels inhibit GnRH and LH secretion via feedback to the brain and pituitary, exacerbating anovulatory conditions. PFAS-induced modifications in Amh and Amhr2 promoter methylation have been observed in murine models, suggesting a possible role for epigenetic reprogramming [39]. Moreover, simultaneous exposure to PFAS and hyperinsulinemia—a common feature in PCOS—may collaboratively enhance Amh expression via IRS-1/AKT-mediated regulation of FOXO1, thus linking metabolic and reproductive dysfunction.

PFASs interfere with the transcriptional function of genes related to steroidogenesis in the ovary, including StAR, CYP11A1, HSD3B1, and CYP19A1, which are precisely regulated by gonadotropin-dependent and intracellular second messenger pathways [18]. PFAS exposure has been shown to inhibit the promoter activity of Cyp19a1 by reducing CREB phosphorylation and hindering SF-1/LRH-1-mediated transactivation. PFASs diminish the quantity of cholesterol that may access mitochondria by destabilising the StAR-VDAC1-TSPO complex [40,41]. This complicates the synthesis of pregnenolone. In TCs, PFASs obstruct LH-induced activation of CYP17A1 and CYP11A1, reducing androgen synthesis and consequently decreasing the substrate pool for aromatisation in GCs. Furthermore, extended exposure to PFASs may modify the intra-ovarian steroid milieu, favouring Δ4-ketosteroids, which induces a pro-inflammatory environment via increased COX2 and PGF2α production, thereby promoting follicular degeneration and the formation of luteinized unruptured follicles [18]. The cumulative disruption of steroidogenesis leads to reduced E2 levels, compromised follicle selection, and heightened activation of apoptotic pathways due to the overexpression of BAX and CASP3, along with the downregulation of BCL2, eventually promoting atresia [42].

In germ cells, PFASs activate PPARγ, which antagonises FSHR-mediated cAMP/PKA signalling and inhibits the function of transcriptional targets such as Fshr, Cyp19a1, Ccnd2, and Inha. Upon activation of PPARγ, the dynamics of nuclear co-regulators are altered by the recruitment of NCoR1 and SMRT, which inhibits gene sets critical for the proliferation of E2 and GC. PFAS-induced PPARγ signalling influences both the PI3K/AKT and MAPK/ERK pathways, complicating the phosphorylation of FOXO3a and BAD. This halts the cell cycle at the G1/S checkpoint and induces mitochondrial apoptosis. Moreover, PFASs diminish the activities of ESR1 and GATA4, critical transcription factors that influence FSH responsiveness and luteinisation. This exacerbates GC dysfunction. Inhibiting FSHR-cAMP signalling disrupts the interaction between BMP and TGFβ within the granulosa cell niche, complicating the collaboration between oocyte and somatic cells, as well as hindering oocyte progression through meiosis, all of which are crucial for the development of the dominant follicle [43,44].

Furthermore, PFASs alter intercellular communication between oocytes and cumulus cells by downregulating connexins such as Cx43, consequently impacting gap junction intercellular communication (GJIC), which is essential for the bidirectional transport of metabolites, ions, and signalling molecules [45]. This communication, enabled by transzonal projections and augmented by GJA1 expression, is crucial for oocyte competence and cumulus cell expansion. PFASs reduce MAPK1/3 activity, resulting in decreased Cx43 phosphorylation, destabilisation of junctional plaques, and reduced permeability [46,47]. Impaired GJIC diminishes the transfer of cAMP and cGMP from cumulus cells to the oocyte, resulting in premature activation of MPF and the initiation of meiosis at varying intervals. The transmission of oocyte-secreted factors such as GDF9 and BMP15 is dysfunctional, resulting in diminished activity of HAS2, PTX3, and TNFAIP6 in cumulus cells [48]. This influences the creation of the extracellular matrix and the initiation of the ovulatory cascade. The intercellular anomalies may clarify the reduced 2PN formation rates and inadequate embryo cleavage potential observed in IVF patients with increased PFAS exposure [49].

PFASs alter the transcription and epigenetic regulation of Amh and Amhr2, thuss influencing the production of AMH. PFASs may either decrease or increase AMH levels, contingent upon the duration of exposure, the concentration present, and the individual’s genotype. This may result in erratic follicular recruitment [50]. In PCOS, marked by elevated AMH levels, PFAS-induced enhancement of AMH expression via FOXO1 and SOX9 stabilisation may intensify granulosa cell arrest and luteinizing hormone hypersecretion [38]. In contrast, DOR phenotypes exhibit downregulation of AMH following PFAS-induced GC apoptosis and a decrease in preantral follicles. PFASs may impair AMH-SMAD signalling by reducing SMAD1/5 phosphorylation, thereby restricting the downstream transcription of ID genes vital for granulosa differentiation [51]. Furthermore, exposure has been linked to altered Amhr2 promoter methylation and histone acetylation patterns, suggesting a prolonged reprogramming of AMH signalling capacity and irregular follicular wave activation dynamics [52].

PFASs induce modifications in DNA methylation at certain loci, histone post-translational alterations, and dysregulation of miRNA at the epigenetic level. These alterations impact the transcriptional mechanisms in germ cells and oocytes. Studies have shown that PFAS exposure leads to hypomethylation at the Fshr, Esr1, and Cyp19a1 promoters, resulting in transcriptional repression or instability [53]. Concurrently, PFASs modulate the activities of DNMT1, DNMT3A, and HDACs, disrupting the chromatin remodelling essential for coordinated gene expression during folliculogenesis. Notably, miRNAs like miR-132, miR-145, and miR-21, which regulate GC formation, steroidogenesis, and apoptosis, exhibit differential expression in the presence of PFASs. This results in anomalous patterns of follicular development [54]. Toxicogenomic studies have demonstrated that the overexpression of CCNB1 and SRD5A1 is associated with increased G2/M arrest and elevated androgen production, respectively, thereby confirming a mechanistic link between PFASs and the pathophysiology of PCOS [55].

Furthermore, variations in miRNA profiles generated by PFASs within FF-derived EVs may serve as paracrine signals that affect GC–oocyte interactions, thereby aggravating follicular dysfunction within the environment. These epigenetic alterations may persist across generations, raising concerns about transgenerational reproductive dysfunction.

PFASs exert diverse effects on female reproduction by disrupting the endocrine system through interference with molecular signalling along the HPG axis, as is shown in Table 1. PFASs suppress Kiss1 and Esr1 at the hypothalamic level, resulting in less pulsatility of GnRH induced by kisspeptin. PFAS suppresses the expression of Gnrhr, Lhb, and Fshb in the pituitary, thus affecting gonadotropin synthesis and secretion via calcium- and MAPK-dependent pathways. Ovarian activities involve the downregulation of Fshr, Star, and Cyp19a1 in granulosa cells, leading to reduced oestradiol production and gonadotropin tolerance. PFASs elevate the expression of Lhcgr, Cyp17a1, and Hsd3b2 in theca cells, resulting in hyperandrogenism, particularly in PCOS. The interruption of Cx43-mediated gap junction communication between granulosa cells and oocytes further impairs follicular growth and oocyte competence.

## 3. Impact on Ovarian Function and Folliculogenesis

The ovary is a hormonally active organ composed of somatic and germ cell compartments that collaboratively regulate folliculogenesis via precisely regulated endocrine, paracrine, and autocrine mechanisms [60]. Exposure to PFASs has been shown to alter multiple levels of this tightly regulated system, leading to dysregulated follicular development, poor steroidogenesis, and accelerated follicular atresia [7]. The effects are mediated by receptor-dependent signalling pathways and receptor-independent physicochemical interactions that undermine GC and TC viability, intercellular communication, and oocyte competence [61]. PFASs engage with intracellular receptors such as PPARs and ERs, alter membrane shape, and disrupt lipid raft domains that contain critical signalling components [30]. This disruption compromises the spatial–temporal fidelity of signalling complexes (including AKT-mTORC1, ERK1/2, and SMADs), thereby destabilising the ovarian niche. Furthermore, PFASs can induce endoplasmic reticulum stress and mitochondrial dysfunction in germ cells by increasing reactive oxygen species and diminishing mitochondrial membrane potential [62]. This initiates the unfolded protein response and intrinsic apoptotic pathways. These cellular lesions impair the survival of growing follicles and disturb the communication between oocytes and neighbouring somatic cells, which is crucial for synchronised follicular maturation [63].

Initial exposure to PFASs during folliculogenesis may interfere with the activation of primordial follicles by altering the PTEN/PI3K/AKT/FOXO3a pathway [64]. Under typical conditions, the activation of PI3K results in the phosphorylation of AKT, which then phosphorylates and inactivates FOXO3a [65]. This enables FOXO3a to exit the nucleus, and inactive follicles to integrate into the growing pool. PFASs impedes PI3K signalling by activating PPARγ or by directly obstructing it via the membrane [66]. This maintains FOXO3a in its active nuclear form. Nuclear FOXO3a interacts with the promoters of genes that facilitate quiescence and apoptosis, including Cdkn1b, Bim, and Gadd45 [67]. This inhibits follicular growth and promotes their demise. PFASs can also enhance the expression of Pten, exacerbating PI3K activity. Concurrently, PFASs diminish the concentrations of growth factors such as Kitl, Bmp15, and Gdf9, which are derived from oocytes and granulosa cells [68]. These factors are crucial for the activation and stabilisation of primordial follicles. Reduced Kitl levels impede c-Kit signalling efficacy in oocytes. This signalling often facilitates the survival and translation of maternal mRNAs by inhibiting Tsc2 and activating mTORC1 via PI3K/AKT [69]. Similarly, the inhibition of Gdf9 and Bmp15 impairs the SMAD2/3 and SMAD1/5/8 signalling pathways, respectively, thus diminishing the proliferation of granulosa cells, the formation of gap junctions, and the support of oocytes [42]. These defects compromise the oocyte–granulosa cell interaction that governs the transition from dormancy to proliferation. The final result is either prolonged follicular dormancy, indicating a reduced recruitment of follicles into the growing follicle pool, or premature activation, followed by atresia, signifying a depletion of the ovarian reserve. Research in animals has shown that exposure to PFASs reduces PF numbers and induces irregular growth wave patterns, indicative of a disrupted PI3K/FOXO3a rheostat [70,71].

Table 2 shows that PFASs impair important molecular checkpoints that control follicle recruitment, growth, steroidogenesis, and survival, which renders folliculogenesis less successful. In the initial stages, the activation of the FOXO3a axis and the suppression of Kitl and Gdf9 limit the shift from dormancy to growth. During the change from secondary to antral, the GC steroidogenic function is decreased due to the downregulation of Fshr and Cyp19a1 and the activation of PPARγ. This leads to inadequate synthesis of E2 and HA. In subsequent phases, diminished expression of Pgr and Areg reduces LH sensitivity, inhibiting ovulation and decreasing the quantity of MII oocytes. PFASs also enhance pro-apoptotic signalling via the mitochondrial pathway, resulting in atresia at various stages of follicular development.

During the transition from primary to preantral follicle stages, PFASs interfere with FSHR expression and function, which are crucial for initiating GC proliferation, E2 generation, and the structural organisation of follicles [31]. PFASs suppresses Fshr transcription through PPARγ-mediated recruitment of HDACs and NCoR1 at its promoter region, thus reducing FSH sensitivity. This leads to inadequate activation of the cAMP/PKA pathway and reduced phosphorylation of CREB, resulting in the downregulation of Cyp19a1, Hsd3b1, Ccnd2, and Inha. Consequently, GCs have diminished mitotic activity, reduced E2 biosynthesis, and decreased differentiation. Furthermore, PFASs attenuate the PI3K/AKT pathway, which collaborates with FSHR signalling to facilitate GC growth by elevating Pten levels and reducing Igf1r levels. This concurrent inhibition prevents the transition from the preantral to the antral stages, where robust development and steroidogenesis are essential [72,73].

PFAS disrupts AMH/AMHR2-SMAD1/5 signalling, influencing FSH sensitivity and regulating the development of preantral follicles. The methylation of the Amhr2 promoter and altered histone acetylation patterns generated by PFASs reduce Amhr2 expression in granulosa cells, limiting subsequent SMAD activation and altering follicle selection [22]. The dysfunction of AMHR2 diminishes the responsiveness of granulosa cells to AMH, disrupting the equilibrium between dormant and growing follicles [74]. Moreover, PFASs diminish Cx43 levels, thereby destabilising GJIC and impeding the transfer of cAMP, cGMP, and pyruvate from CCs to the oocyte. This disturbance in gap junction-mediated metabolic exchange results in abnormal oocyte development and compromised cumulus cell growth. Inadequate paracrine signalling from the oocyte, including Gdf9 and Bmp15, intensifies the transcription of cumulus cell-specific genes (Has2, Tnfaip6, Ptx3), thereby undermining the extracellular matrix and modifying the composition of follicular fluid [75,76].

In antral follicles, exposure to PFASs exacerbates these issues. The transition to the antral phase requires increased metabolic activity and heightened hormonal sensitivity. The control of Esr1 and Esr2 by PFASs inhibits oestrogen receptor-dependent transcriptional activity, affecting genes related to follicular development and vascularisation (Vegfa, Lhcgr, Fshr). PFASs induce oxidative stress responses via Nrf2-Keap1 signalling and elevate markers of mitochondrial dysfunction (Drp1, Fis1, Pink1), resulting in increased ROS levels. This induces apoptosis by increasing the permeability of the mitochondrial outer membrane and releasing cytochrome c [77,78]. Elevating the levels of Bax, Casp3, and Trp53 while diminishing Bcl2 initiates the intrinsic apoptotic cascade, accelerating germ cell death and follicular depletion [79]. Follicles exposed to PFASs exhibit diminished levels of Vegfa and angiopoietin, impeding the follicle’s ability to regenerate its blood vessels and transport nutrients.

In the preovulatory phase, the dominant follicle demonstrates increased sensitivity to LH due to the overexpression of Lhcgr in mural granulosa cells, a process driven by E2-induced Esr1 activation and FSHR-mediated cAMP/CREB signalling [80,81]. PFASs impede this priming process by suppressing the transcription of Esr1 and Lhcgr, leading to reduced LH sensitivity. As a result, the ability of GCs to begin a transcriptional response to LH surge is diminished [82]. This involves the impaired production of the EGF-like ligands Areg, Ereg, and Btc, which are crucial mediators of LH-induced cumulus growth via EGFR transactivation in cumulus cells. PFAS exposure reduces the expression of Areg and Ereg, thereby limiting the subsequent activation of MAPK1/3 and ERK1/2. This then affects the phosphorylation of CREB, p38, and RSK1, which is crucial for ovulatory gene transcription [83,84].

When this signalling cascade is disrupted, critical genes associated with ovulation, including Ptgs2, Pgr, Adamts1, and Has2, exhibit diminished functionality. The suppression of Ptgs2 by PFASs impedes PGE2 synthesis, which is essential for follicular rupture, vascular remodelling, and cumulus development [85]. Concurrently, the downregulation of Pgr results in compromised proteolytic remodelling of the follicular wall, while the inhibition of Adamts1 obstructs ECM cleavage. Furthermore, diminished levels of Has2 in cumulus cells result in reduced hyaluronic acid synthesis, thereby impeding cumulus expansion and oocyte release [86]. These molecular aberrations lead to ovulatory failure and correspond with LUFS, which is linked to ongoing environmental endocrine disruption.

PFASs also alter the responsiveness of the oocyte to ovulatory cues. Downregulation of Cx43 impairs gap junction intercellular communication, hindering the prompt transport of cAMP and cGMP from the oocyte. This prematurely activates MPF, resulting in unstable meiotic growth [87]. PFASs alter the cytoplasmic competence of oocytes by diminishing mitochondrial membrane potential and ATP levels, while increasing reactive oxygen species and mitochondrial fragmentation via Drp1 overactivation. These alterations diminish spindle stability and chromosome alignment, thus increasing the probability of aneuploidy [88]. From a structural perspective, PFAS-exposed follicles fail to achieve full cumulus expansion and display partial follicular rupture, resulting in the entrapment of oocytes and compromised corpus luteum development. Transcriptomic analyses of preovulatory follicles influenced by PFASs indicate that ovulatory cluster genes and extracellular matrix remodelling components are downregulated, thus supporting the hypothesis that the LH–EGF–MAPK axis has been compromised.

PFASs influence every aspect of the ovulatory process: hormonal response (Lhcgr, Esr1), signal transmission (Areg, Ereg, Mapk), enzymatic activation (Ptgs2, Adamts1), and structural remodelling (Has2, Tnfaip6). This results in a complicated ovulatory dysfunction that complicates conception, despite normal follicular development.

## 4. PFASs and Polycystic Ovary Syndrome (PCOS)

PCOS is a complex endocrine disorder characterised by hyperandrogenism (HA), ovulatory dysfunction (OAD), and polycystic ovarian morphology (PCOM). Increasing research suggests that individuals with PCOS may exhibit heightened sensitivity to environmental endocrine-disrupting chemicals (EDCs) such as PFASs, due to their existing dysregulated metabolism, hormones, and genetics [89]. The convergence of the essential biological traits of PCOS—insulin resistance, hyperandrogenism, and chronic inflammation—with the recognised mechanisms of PFAS toxicity supports a synergistic pathogenic hypothesis. Cross-sectional and case-control studies have consistently shown elevated serum concentrations of PFOS, PFOA, and PFHxS in women with PCOS compared to controls. These chemicals are positively correlated with increased T, DHEAS, and the LH/FSH ratio, as well as altered SHBG levels and elevated body mass index (BMI), suggesting systemic endocrine disruption [90]. The accumulation of PFASs in adipose tissue and ovarian stroma may exacerbate the hyperandrogenic and pro-inflammatory milieu characteristic of the PCOS phenotype. Due to their prolonged biological half-lives, PFASs may function as enduring enhancers of the PCOS cascade, exacerbating hormonal imbalance and metabolic rigidity even after exposure ceases [16].

PFASs interfere with critical regulators of steroidogenesis and folliculogenesis at the molecular level by engaging the PPARγ and ER pathways. In TCs, PFASs activate PPARγ and enhance Lhcgr expression, leading to increased LH sensitivity and the overexpression of Star, Cyp17a1, and Hsd3b2 [7]. These enzymes facilitate the conversion of cholesterol into A4 and subsequently T, thus elevating androgen levels in the ovaries. Concurrently, PFASs inhibit Esr1-mediated negative feedback mechanisms, resulting in elevated testosterone levels. This disrupts the steroidogenic equilibrium and elevates the A4:T:E2 ratios commonly observed in PCOS. In granulosa cells, PFASs impede FSHR–cAMP–PKA–CREB signalling by reducing Fshr transcription and CREB phosphorylation, thereby diminishing the activity of Cyp19a1 and aromatase [91]. The diminished conversion of A4 to E2 intensifies the HA condition and obstructs the E2-induced activation of LH surge initiators, such as Areg, Pgr, and Ptgs2. Moreover, reduced E2 levels impede the activation of Esr1 and Esr2, thus establishing a feedforward cycle of oestrogen deficit [92]. The resultant situation is a diminished follicular state in which excessive androgens inhibit granulosa cell growth, retard oocyte maturation, and accelerate follicular atresia. This resembles the persistent anovulatory phenotype associated with PCOS. Besides steroidogenesis, PFAS exposure in PCOS models has been associated with increased expression of Insr and Irs1, as well as atypical serine phosphorylation, which reduces insulin sensitivity in theca and stromal cells. This further activates Cyp17a1 via Foxo1-dependent derepression, linking metabolic issues with reproductive concerns. Furthermore, PFAS-mediated activation of Tnfα, Il6, and Nfkb intensifies ovarian inflammation, impairs angiogenesis, and elevates ROS generation, resulting in the modified stromal architecture and fibrosis commonly seen in PCOS ovaries [93].

Along with messing with hormones and metabolism, PFASs also mess up the local ovarian regulatory network that controls early follicular development. The AMH–AMHR2 system is one of the main targets [7]. In polycystic ovary syndrome (PCOS), AMH levels are significantly higher due to an increased number of granulosa cells (GCs) and enhanced transcriptional activity mediated by Foxl2, Sox9, and Gata4 [94]. PFAS exposure exacerbates this dysregulation by causing epigenetic silencing of Amhr2 via DNMT1-mediated CpG methylation and diminished H3K9ac, resulting in compromised SMAD1/5/8 signalling. This desensitisation of GCs to AMH disturbs the equilibrium between follicular dormancy and development, promoting the accumulation of preantral and early antral follicles that exhibit an inadequate FSH response. The result is a messed-up follicular hierarchy, with no transition into the dominant phase [95].

At the same time, PFASs change important regulators of cell cycle progression, which makes GCs grow and survive less well. The downregulation of Ccnd2, Ccne1, and E2f1, along with the overexpression of Cdkn1a and Trp53, suggests that there is a P53-dependent checkpoint arrest.

These changes are made worse by lower Igf1r signalling and AKT hypoactivation, which makes anabolic responses even less likely and encourages FOXO-mediated pro-apoptotic gene expression (Bim, Faslg) [96]. There is also stress in the mitochondria, as seen by lower levels of Sod2, higher levels of Drp1, and higher levels of mtROS generation. This activates intrinsic apoptotic pathways and lowers ATP stores in GCs. In all, this limits the vitality of follicles and adds to the atretic character found in PCOS models that have been exposed to PFASs [97].

PFASs also mess up communication between cells in the follicular unit. When Cx43 (also known as Gja1) is turned down, the GJIC between GCs and oocytes is reduced. This makes it harder for cGMP and cAMP to move between them, which is needed for meiotic arrest and metabolic support [7]. Simultaneously, oocyte-derived components Gdf9 and Bmp15, which are essential for CC proliferation and differentiation, are inhibited through reduced SMAD2/3 phosphorylation and epigenetic repression of their upstream promoters [98]. These deficiencies hinder the transcription of downstream cumulus-specific genes, including Has2, Tnfaip6, Ptx3, and Hyal2, resulting in poor ECM remodelling and compromised cumulus expansion. These molecular occurrences make the oocyte microenvironment insufficiently supportive for cytoplasmic and nuclear maturation, which lowers the ability to develop.

The structural outcome of these molecular injuries is a fibrotic, hypercellular ovarian cortex characterised by elevated collagen deposition, stromal hyperplasia, and aberrant vasculature, as evidenced by the upregulation of Col1a1, Tgfβ1, and Vegfa in PFAS-exposed PCOS ovaries [99]. Immunohistochemistry shows that small antral follicles stay around longer without or with delayed atresia, greater vascular resistance, and less follicular fluid volume. These are all things that make ovulation harder and break the endocrine feedback loop with the HPG axis [100]. Consequently, PFASs exacerbate the AMH–FSHR disconnection that characterises PCOM and introduce further dysfunction through mechanisms such as apoptosis, oxidative stress, and transcriptome remodelling. This biochemical disturbance fortifies the anovulatory condition and exacerbates the persistent reproductive failure characteristic of PCOS.

PFASs exert lasting effects on ovarian function at the epigenetic level by modifying DNA methylation, histone alterations, and non-coding RNA networks. In polycystic ovarian syndrome (PCOS), where epigenomic instability affects Fshr, Cyp19a1, Esr1, and Igf1, PFASs intensify transcriptional repression through DNMT3A-mediated CpG hypermethylation and global hypoacetylation of H3K9 and H4K16 [101]. These alterations impede access to promoters and diminish the body’s sensitivity to gonadotropins and intraovarian growth hormones. For example, diminished amounts of H3K27ac at the Fshr locus result in reduced cAMP/PKA signalling, thus impeding CREB’s ability to activate and produce aromatase downstream. Methylation of the Amhr2 and Esr2 loci inhibits their transcription, diminishing the synergy between AMH and FSH and the feedback regulation of E2, respectively [102].

PFASs alter chromatin remodelling by interfering with histone acetyltransferases (such as p300/CBP) and histone deacetylases (such as HDAC4 and HDAC9). This renders the chromatin more transcriptionally restrictive. This epigenetic repression facilitates the survival of blocked follicles and complicates the collaboration between oocytes and granulosa cells [103].

Concurrently, PFAS exposure diminishes the expression of TET1 and TET3, thereby affecting active demethylation processes in the oocyte and GC genomes throughout folliculogenesis [104].

These enzymes are crucial for sustaining meiotic gene expression and mitochondrial equilibrium; their inhibition has been linked to disrupted spindle assembly and the accumulation of DNA damage.

In the ncRNA landscape, PFASs modulate miRNA expression profiles that are already altered in PCOS. The overexpression of miR-145, miR-21, and miR-27a has been recorded, resulting in the suppression of targets such as Ccnd2, Sod2, Ppargc1a, and Esr1, which leads to impaired proliferation, oxidative dysregulation, and oestrogen insensitivity. Conversely, PFASs inhibit miR-132 and miR-126, which are microRNAs that typically facilitate GC survival and angiogenesis via Igf1r and Vegfa. These alterations exacerbate follicular dysfunction by modifying critical regulatory circuits and enhancing anti-apoptotic resistance, preventing the elimination of defective follicles. Furthermore, recent studies suggest that PFAS-induced miRNA dysregulation may be transmitted between generations via altered oocyte cytoplasmic content and cumulus-derived exosomes. In animal models, in utero exposure to PFHxS and PFOA led to persistent alterations in ovarian miR-let7, miR-29, and miR-34 family members in female offspring, associated with reproductive abnormalities and altered ovarian gene networks. These findings raise concerns about the transgenerational inheritance of reproductive risks due to PFAS-induced epigenomic reprogramming.

## 5. PFASs and IVF Outcomes

IVF is most effective when the endocrine system functions harmoniously, follicles mature appropriately, oocytes exhibit superior quality, and the endometrium is prepared for implantation. Increasing epidemiological and translational evidence indicates that PFAS exposure negatively impacts ART outcomes through systemic endocrine disturbance and localised ovarian injury. Multiple clinical studies have demonstrated inverse relationships between serum PFOS/PFOA levels and critical IVF parameters, including the quantity of retrieved oocytes, MII oocyte rate, fertilisation rate, percentage of high-quality embryos, and live birth rate (LBR). The results retain significance after adjusting for age, BMI, AMH, and stimulation protocol [17].

At the ovarian level, PFASs diminish COS efficacy by downregulating Fshr transcription via hypermethylation and HDAC-mediated chromatin condensation, leading to decreased cAMP/PKA/CREB signalling. Reduced phosphorylation of CREB impedes the activation of Star, Cyp11a1, and Cyp19a1, thereby inhibiting the conversion of cholesterol into P and subsequently E2. This diminishes the synthesis of glucocorticoids, reduces the expression of Lhcgr, and impedes the maturation of preovulatory follicles [105]. The developing follicles exhibit insufficient contact between the granulosa and theca, along with suboptimal antral expansion. The downregulation of Hsd3b1 further restricts Δ4 pathway activation, intensifying oestrogen deficit despite a normal follicular count.

The diminished steroidogenic capacity leads to lower serum E2 levels per follicle on the day of the hCG trigger, indicating a functional disjunction between follicle count and endocrine competence [106]. This is associated with an increased requirement for gonadotropins, extended stimulation cycles, and a reduced number of follicles reaching a diameter of 17 mm or greater.

PFASs enhance the activity of Socs3 and Tnfa, undermining the synergy between FSH and IGF1, and induce GC apoptosis by inhibiting STAT3. PFASs alter the action of Igf1r–AKT, undermining the anabolic support essential for oocyte cytoplasm development and granulosa cell proliferation [6].

Impaired mitochondrial function in the follicular niche exacerbates hormone resistance. PFASs elevate mtROS by inhibiting Sod2, Gpx4, and Ucp2, while increasing Drp1 and disrupting mitochondrial fission and fusion processes [107]. This impedes ATP synthesis, activates AMPK, and inhibits the proliferation of follicular cells. Mitochondrial damage impedes the exchange of metabolic information between oocytes and granulosa cells by destabilising NAD+/NADH and acetyl-CoA pools [108]. These pools are crucial for histone acetylation and the resumption of meiosis. Ultimately, impaired steroidogenesis inadequately prepares the endometrium. Blunted E2 exposure suppresses the expression of Esr1, Pgr, and Lif, which are critical for stromal proliferation and receptivity. The insufficient elevation of E2 interferes with the feedback-dependent LH surge mimicked by GnRHa or hCG stimulation, resulting in asynchronous follicular rupture or premature luteinisation (LUF) [109]. The accumulated deficits lead to reduced oocyte production, lower maturation rates, and disrupted endometrial–embryo communication, ultimately compromising clinical pregnancy rates (CPRs) and live birth rates (LBRs).

Moreover, PFASs impede follicular steroidogenesis and reactivity to COS, while substantially compromising oocyte quality by disrupting cytoplasmic and nuclear maturation. PFAS exposure in oocytes increases mtROS and decreases MMP by elevating Drp1 and reducing Opa1 and Mfn2 [110]. This complicates the fusion of mitochondria and the generation of new ones. This leads to ATP depletion, a reduction in mtDNA copy number, and compromised organelle distribution—factors essential for meiotic growth and spindle assembly [111]. Disruption of mitochondrial dynamics also reduces the expression of Pgc1α, Tfam, and Polg, thereby impairing the ability of mitochondria to replicate and transcribe genes during the GV–MII transition.

The integrity of the spindle is markedly compromised, as PFASs disrupt the production of α/β-tubulin heterodimers and centrosomal orientation, through the downregulation of Tubb8, Aurka, and Plk1. These alterations result in errors in chromosomal congression, delayed chromosomes, and anaphase bridges, thus increasing the incidence of aneuploidy. Immunostaining of oocytes from PFAS-exposed models reveals disrupted meiotic spindles, pericentric misalignment, and premature SAC deactivation. This is partially due to the reduced levels of Bub1 and Mad2l1. Cytoplasmic immaturity is characterised by an altered distribution of cortical granules and a reduction in Nlrp5, Zar1, Mater, and Ooep—maternal effect genes crucial for zygote activation and the early phases of embryogenesis. These oocytes have inadequate calcium oscillatory responses during ICSI and a reduced capacity for pronuclear growth. Simultaneously, PFASs elevate indicators of autophagy and apoptosis (Becn1, Casp3, Bax) while diminishing markers of anti-apoptosis (Bcl2), thus promoting cellular degeneration.

### CC Dysfunction Contributes to This Detrimental Microenvironment

The inhibition of Cx43 and Gja1 by PFASs reduces GJIC, obstructing the transport of cAMP, amino acids, and antioxidants [112]. Decreased levels of Has2, Tnfaip6, and Ptx3 result in the disorganisation of the extracellular matrix, thereby diminishing the efficacy of cumulus expansion. This undermines the mechanical support and signal transmission essential for synchronised maturation [113]. These alterations collectively impede communication between oocytes and cumulus cells, resulting in asynchronous meiotic and cytoplasmic maturation. PFASs diminish cleavage rates, augment blastomere asymmetry, and impair compaction in the embryo, as indicated by the altered expression of Cdx2, Oct4, and Sox2. Both the ICM and TE lineages have an elevated DNA fragmentation index, enlarged mitochondria, and a reduced cell count in blastocysts. Embryos from populations exposed to PFASs have reduced hatching potential and inadequate in vitro growth, which is associated with lower implantation rates and increased miscarriage risk [114].

PFASs significantly diminish endometrial receptivity by disrupting hormone signalling, epithelial–stromal interactions, angiogenic equilibrium, and immunological homeostasis during the window of implantation [115].

In physiological contexts, synchronised E2 and P signalling via Esr1 and Pgr modulates the expression of downstream targets essential for stromal decidualisation, epithelial receptivity, and blastocyst adhesion [116]. PFAS exposure downregulates both Esr1 and Pgr, leading to reduced transcription of Hoxa10, Hoxa11, Fkbp5, and Ihh, all of which are crucial for the successful conversion of stromal fibroblasts into decidual cells. The CpG methylation in promoter areas and the reduced accumulation of H3K4me3 are responsible for this hormonal desensitisation. This ultimately deactivates essential implantation genes. Furthermore, PFASs impair epithelial responsiveness by altering cell polarity and the synthesis of adhesion molecules. Downregulation of Muc1, Lif, Ostf1, and Gp130 impedes the uterine luminal surface’s ability to locate and adhere to the opposing blastocyst. The inadequacies are intensified by altered cytoskeletal organisation, caused by the decrease in Ezrin, Actn1, and Rdx, leading to impaired apical–basal architecture. PFASs inhibit the functioning of Itgav, Itgb3, and Fn1, thus obstructing integrin-mediated adhesion and complicating the docking process for embryos.

In the decidual compartment, PFASs inhibit the differentiation of stromal cells by downregulating Bmp2, Hand2, Prl, Wnt4, and Igfbp1, which are essential mediators of decidual maturation. Phosphorylation of Stat3 is markedly reduced, hindering Lif-induced signalling, while aberrant activation of AhR leads to the overexpression of Cyp1a1 and Tgfb1, fostering a pro-fibrotic condition that inhibits proper decidualisation. This is associated with reduced Foxo1 nuclear localisation and the downregulation of Hbegf and Il11, which limits the interaction between the blastocyst and the endometrium.

Angiogenic remodelling is adversely affected as well. Exposure to PFASs diminishes the transcription of Vegfa, Angpt1, Flt1, and Kdr, resulting in insufficient spiral artery remodelling and poor neovascularisation. Endothelial dysfunction is exacerbated by reduced Nos3, elevated Edn1, and suppressed Hif1α transcription, leading to impaired oxygen transport and localised hypoxia [117]. These conditions alter the secretion of chemicals by stromal cells and impede the migration of leukocytes to the site of immune–vascular alignment.

The dysregulation of cytokine and chemokine networks generated by PFASs further destabilises the immunological landscape of the endometrium. Inhibiting Il15, Cxcl12, and Ccl5 impedes the mobility of NK cells within the uterus [118]. Conversely, the elevation of Il6, Tnf, and Il1b intensifies NF-κB signalling and fosters an environment detrimental to implantation. Owing to Irf5 overexpression and Arg1 suppression, there is a reduction in DC-SIGN+ dendritic cells, and macrophage polarisation shifts towards a pro-inflammatory M1 phenotype [119]. These immunological disruptions reduce embryo tolerance and increase the likelihood of early pregnancy loss.

PFASs alter the methylome and histone configuration of endometrial stromal and epithelial cells at the epigenetic level. The modified expression of Dnmt1, Dnmt3b, Hdac4, Hdac9, and Tet2 influences the accessibility of promoters for genes associated with receptivity, particularly Lif, Esr2, Gp130, and Pgr. A global reduction in H3K27ac and an elevation in H3K9me2 marks inhibit chromatin regions critical for decidualisation. PFASs disrupt endometrial miRNAs. Excessive levels of miR-200c, miR-22, and miR-181a inhibit Zeb1, Pgr, and Muc1, whereas insufficient levels of miR-126, miR-30b, and miR-145 impede angiogenesis and stromal development by diminishing the regulation of Vegfa and Gja1 [120].

These several issues collectively render the endometrium unresponsive. This occurs because of its resistance to hormones, emission of pro-inflammatory signals, inability to develop blood vessels, and suppression of crucial genes via epigenetic mechanisms. In ART contexts, these modifications manifest as diminished endometrial thickness, reduced implantation rates, compromised hCG elevation upon transfer, and an increased incidence of biochemical pregnancies or early miscarriages.

## 6. Clinical and Public Health Implications

PFASs, as prominent EDCs, have introduced a new dimension to the evaluation and management of female infertility. While there has been great advancement in grasping ovarian ageing, hormonal imbalances, and embryological viability, the influence of environmental toxicants, particularly PFASs, is still inadequately incorporated into clinical reproductive medicine. Their physicochemical stability, bioaccumulation in lipid-rich tissues, and interaction with hormone receptors and epigenetic regulators impose a continual and frequently unrecognised impact on reproductive health. There is more and more proof that PFASs are bad for ovarian reserve, endometrial receptivity, and the quality of oocytes and embryos. However, clinical protocols infrequently evaluate this exposure. In a context where unexplained infertility is on the rise and IVF outcomes remain unsatisfactory for many women, PFASs represent a controllable yet largely overlooked etiological component.

From a clinical perspective, multiple prospective and retrospective studies have established a consistent link between increased serum PFAS levels and compromised ART outcomes. PFOS, PFOA, and PFHxS concentrations are specifically related to fewer retrieved oocytes, reduced rates of MII oocytes, decreased fertilisation ratios, and poor blastocyst development. Even after correcting for covariates, including age, BMI, baseline AMH, and total gonadotropin dose, live birth rates (LBRs) and cumulative pregnancy rates (CPRs) are still negatively influenced. These data demonstrate that PFASs interfere with ovarian response to COS by downregulating FSHR and aromatase genes, reducing oestrogen synthesis per follicle, and changing the follicular microenvironment. The effect is not only on number but also on quality: PFASs damage mitochondria, mess with spindle dynamics, and make oocytes’ cytoplasm immature. Consequently, women receiving IVF may demand repeated rounds and increased dosages, potentially leading to inferior outcomes if their environmental burden continues unchecked.

The absence of PFAS exposure integration in infertility screening implies both diagnostic and systemic inadequacies. There are ways to measure the amount of PFASs in blood or follicles; however, they are not standardised in clinical labs, and there are no clear reference values for reproductive outcomes. Moreover, modern patient histories often overlook environmental exposure unless specifically addressed. However, women in specific professions (e.g., firefighting, chemical production, textile treatment) or geographic areas (e.g., proximity to contaminated water sources) may possess substantially increased PFAS levels. Preconception counselling should encompass lifestyle and environmental assessments, accompanied by pragmatic suggestions for exposure mitigation—such as transitioning to PFAS-free cookware, purifying drinking water, and reducing the intake of fast food or packaged items recognised for leaching PFAS compounds. These therapies may be especially advantageous for individuals with RIF, PCOS, diminished ovarian reserve, or inadequate embryo development, as PFAS toxicity may aggravate pre-existing malfunction.

PFAS impacts extend beyond a single patient or cycle; they will influence future generations as well. Transplacental transfer of PFASs results in detectable levels in cord blood, and PFASs are also formed in breast milk, resulting in chronic low-level exposure during infancy. Research utilising animal models indicates that prenatal exposure to PFASs can hinder primordial follicle development, delay puberty, and affect HPG axis signalling in adults. Data from human cohorts reveal the same patterns, with early-life PFAS exposure connected to earlier menarche, irregular menstrual cycles, and symptoms of premature reproductive ageing. The potential for epigenetic programming of reproductive dysfunction—transmitted across generations—underscores the significance of integrating PFASs into reproductive toxicology and public health frameworks. If unaddressed, this environmental burden could subtly alter the reproductive patterns of individuals in industrialised cultures.

Regulation of PFASs is still not strong enough or consistent enough at the policy level. The Stockholm Convention has limited the use of several compounds, such as PFOS and PFOA. However, thousands of PFAS analogues that are structurally identical are still employed, and many of them have the same or even higher bioactivity. Present toxicity testing guidelines insufficiently address reproductive endpoints, notably those relevant to oocyte quality, implantation, and embryonic development. Furthermore, PFASs operate through non-monotonic dose–response curves and synergistic effects that circumvent traditional toxicological thresholds. Regulatory organisations need to employ more advanced models of endocrine disruption, include molecular data in risk assessments, and give more weight to cumulative exposure from food, drinking water, and consumer products. National infertility surveillance projects ought to address the incorporation of environmental biomonitoring and the connection of PFAS exposure with IVF registries and birth cohort data.

In sum, PFASs are no longer theoretical risks—they are tangible, measurable, and mechanistically tied to reproductive damage. Their repercussions include endocrine resistance, mitochondrial malfunction, epigenetic silencing, and immunological imbalance. Including PFASs in the therapeutic dialogue about infertility is a vital method to minimise iatrogenic failure, improve ART outcomes, and protect reproductive potential for future generations. Moving forward, reproductive endocrinologists, embryologists, toxicologists, and public health professionals must align efforts to develop screening tools, mitigation approaches, and policy responses that reflect the environmental reality of 21st-century reproduction.

In Table 3, we provide a summary of the main PFAS-induced changes in female reproductive physiology to make it easier to connect complex molecular pathways with clinical results. The table shows how certain molecular events, such as hypothalamic hormonal suppression, granulosa cell dysfunction, epigenetic changes, and inflammation, are linked to different reproductive phenotypes, such as delayed puberty, poor ovarian response, lower endometrial receptivity, and a higher risk of infertility. This table provides a translational view that can help doctors and researchers understand how PFAS exposure affects fertility-related endpoints in many ways by putting molecular insights together with clinical outcomes.

## 7. Conclusions and Future Directions

PFASs have become powerful and pervasive EDCs that have deep, multi-layered effects on female reproductive biology. Their lipophilic nature and metabolic persistence enable systemic transit and accumulation in hormone-sensitive organs, where they interfere with HPG signalling at the hypothalamus, pituitary, and ovarian levels. PFASs downregulate key transcriptional regulators such Esr1, Pgr, Star, Cyp19a1, Hsd3b1, and Fshr. This makes COS less sensitive, stops E2 from being created, and stops GCs from distinguishing. Some of the impacts are a decreased follicular yield, a weaker sensitivity to outside gonadotropins, and a higher resistance to gonadotropins. Furthermore, PFASs affect CC–oocyte interaction by modifying GJIC via downregulation of Cx43, leading to asynchronous nuclear and cytoplasmic maturation, decreased MII oocytes, and lower developmental competence, ultimately reducing fertilisation and blastulation rates in ART.

Concurrently, PFAS-induced activation of PPARγ suppresses FSHR–cAMP/PKA/CREB signalling, leading to decreased Cyp19a1 expression and lower aromatisation efficiency in granulosa cells. Simultaneously, disruption of the PI3K/AKT/FOXO3a axis maintains oocytes in a dormant or apoptotic state by preserving nuclear FOXO3a localisation and reducing Kitl, Bmp15, and Gdf9 expression. These routes together promote early follicular atresia, fewer antral follicles, and reduced OR. The effects on the endometrium are just as serious: PFASs reduce Hoxa10, Hoxa11, Lif, Itgb3, Bmp2, and Igfbp1, which makes it harder for the endometrium to decidualise, for epithelial polarity to form, and for stromal–embryonic synchronisation to occur during WOI. Moreover, interfering with Stat3 and AhR pathways shifts the uterine milieu toward an anti-receptive state, reducing LBR and implantation probability in IVF.

At the immunological and vascular interface, PFASs induce a pro-inflammatory endometrial phenotype by upregulating Tnf, Il6, and Il1b, while simultaneously inhibiting tolerogenic signals such Ccl2 and Cxcl12, thereby impeding leukocyte recruitment and stromal–immune communication. At the same time, angiogenic imbalance arises due to the inhibition of Vegfa, Flt1, and Angpt1, as well as the dysregulation of Hif1α/Nos3. This leads to insufficient remodelling of the spiral artery, increased resistance in the uterine artery, and localised hypoxia. These vascular alterations impede the endometrium’s ability to receive blood and nutrients during implantation and early gestation.

Women with PCOS or DOR appear particularly vulnerable to PFAS poisoning, since their baseline hormonal, metabolic, and inflammatory milieu predisposes them to additive or synergistic disruption. In TCs, PFASs upregulate Cyp17a1, Hsd3b2, and Lhcgr, which enhances the synthesis of androgens. At the same time, it downregulates Cyp19a1 in GCs, which raises the A4:T ratio and makes HA worse. This leads to ovulatory arrest, underdeveloped follicles, and poor endometrial priming. Furthermore, PFASs change systemic insulin resistance by altering insulin receptor signalling and adipokine synthesis, aggravating the pathogenesis of PCOS.

PFASs cause heritable alterations in DNMT1, TET2, HDAC1/2, and non-coding RNAs through epigenetics. Differential methylation of Hoxa10, Esr2, Lif, and Zeb1 is related to reduced receptivity and lower endometrial plasticity. Dysregulated miRNAs, such as miR-22, miR-200c, miR-181a, and let-7a, block genes necessary for oocyte competence (Gdf9, Bmp15, Sohlh2) and endometrial function (Muc1, Igfbp1, Pgr), leading to implantation failure and early pregnancy loss. These epigenetic markers may survive beyond the reproductive phase, consequently influencing fertility trajectories across generations.

In future studies, it is crucial to prioritise the addition of PFAS monitoring in prospective IVF registries, in conjunction with a thorough assessment of follicular fluid and serum biomarkers. High-throughput toxicogenomic platforms and single-cell omics could elucidate PFAS-induced cell-type-specific transcriptome and epigenomic changes in the ovary and endometrium. It is vital to concentrate on PFAS–miRNA–chromatin remodelling interactions in CCs, GCs, and stromal fibroblasts, since these may operate as predictive biomarkers for ART prognosis. Clinical practices must adapt to incorporate environmental risk assessment and counselling during preconception visits, particularly for high-risk populations (e.g., PCOS, idiopathic RIF, or previous IVF failure).

In terms of regulations, the current systems are still out of date and do not take into account subfertility, oocyte competence, or implantation endpoints when evaluating chemical safety. Regulatory agencies need to widen hazard identification to cover cumulative endocrine-disrupting chemical (EDC) load, low-dose non-monotonic effects, and exposure during important reproductive times. We need to work together on an international basis to set consistent exposure thresholds, limit high-risk PFAS subtypes, and pay for environmental research that emphasises fertility. It is crucial for REI specialists, environmental toxicologists, epigeneticists, and public health policymakers to collaborate across disciplines to establish a translational infrastructure that connects exposure science with reproductive repercussions that can be acted on.

In conclusion, PFASs constitute a widespread and alterable threat to female fertility and the success of assisted reproductive technology (ART). Their impacts can be traced at the molecular level, quantified in clinical settings, and have social ramifications. We can only minimise the reproductive impacts of PFASs in the present and future generations by combining translational research, preventive healthcare practices, and policy changes based on evidence.

## Figures and Tables

**Table 1:** Molecular targets of PFASs across the hypothalamic–pituitary–gonadal (HPG) axis.

Axis Level	Molecular Target	PFAS Effect	Disrupted Signalling Pathway	Functional Outcome	References
Hypothalamus	*Kiss1*, *GPR54*, *Esr1*	Downregulation of *Kiss1* and *Esr1* expression in ARC and AVPV neurons; reduced GPR54 activation	ERα/Kisspeptin GnRH axis	Reduced GnRH pulsatility and delayed pubertal onset	Trevisan et al., 2018; Roseweir et al., 2008 [56,57]
Hypothalamus	*GnRH* neurons	Attenuated pulsatile GnRH release; altered neuronal firing	Glutamate/GABA imbalance; reduced *Npy* and *Tac3* expression	Blunted LH surge; disrupted ovarian cycling	Liu et al., 2022 [41]
Pituitary	*Gnrhr*, *Lhb*, *Fshb*	Suppressed GnRH receptor and β-subunit gene expression	GnRH–Ca^2^⁺–PKC and MAPK pathways	Reduced LH and FSH secretion; impaired ovulatory trigger	Kim and Lawson 2015 [58]
Pituitary	*Pitx1*, *Egr1*	Downregulation of key transcription factors for gonadotropin biosynthesis	GnRH-induced nuclear signalling	Inadequate gonadotropin production	Zhang et al., 2024 [14]
Ovary (GCs)	*Fshr*, *Cyp19a1*, *Star*, *Hsd3b1*	Decreased receptor density and steroidogenic enzyme expression	cAMP/PKA/CREB, FSHR-mediated signalling	Reduced estradiol biosynthesis; poor response to COS	Zhang et al., 2024; Liu et al., 2022 [14,41]
Ovary (TCs)	*Lhcgr*, *Cyp17a1*, *Hsd3b2*	Upregulated expression of androgenic enzymes (especially in PCOS)	PKA activation, loss of negative feedback by *Esr1*	Elevated A4 and T levels; exacerbated HA	Clark et al., 2024, Comim et al., 2013 [6,59]

This table outlines the key molecular targets and signalling pathways affected by per- and polyfluoroalkyl substances (PFASs) across the hypothalamic–pituitary–gonadal (HPG) axis in females. Essential modifications are enumerated by anatomical level, target molecules (hormones, receptors, transcription factors), PFAS-induced effects, impaired signalling pathways, functional reproductive consequences, and pertinent references.

**Table 2:** PFAS-mediated molecular alterations in folliculogenesis.

Follicular Stage	Cell Type	Key Molecular Targets	PFAS-Induced Alterations	Disrupted Pathways	Functional Outcome
Primordial → Primary	Pre-GCs, oocyte	*Pten*, *Foxo3a*, *Kitl*, *Gdf9*, *Bmp15*	↑ Nuclear *FOXO3a*, ↓ *Kitl/Gdf9* expression	PI3K/AKT/FOXO3a	Inhibited activation, follicle dormancy or apoptosis
Primary → Secondary	GCs, oocyte	*Cx43*, *Gja1*, *Zp3*, *Tnfaip6*	↓ Connexin expression, impaired GJIC	GJIC/cAMP/PKA	Disrupted GC–oocyte communication; poor cytoplasmic maturation
Secondary → Antral	GCs, TCs	*Fshr*, *Cyp19a1*, *Star*, *Hsd3b1*, *Lhcgr*, *Cyp17a1*	↓ *Fshr/Cyp19a1*, ↑ *Lhcgr/Cyp17a1*	cAMP/PKA/CREB, PPARγ, steroidogenesis	Reduced E2, ↑ A4/T, impaired antral expansion
Antral → Preovulatory	GCs, oocyte	*Pgr*, *Esr1*, *Areg*, *Ereg*, *Has2*	↓ LH-responsiveness, impaired cumulus expansion	LH/EGFR, ERK1/2	Ovulatory failure; reduced MII rate
Atresia (any stage)	GCs	*Bax*, *Bcl2*, *Casp3*, *Foxo1*	↑ Pro-apoptotic, ↓ anti-apoptotic signals	Mitochondrial apoptosis pathway	Accelerated follicular atresia
Follicular Stage	Cell Type	Key Molecular Targets	PFAS-Induced Alterations	Disrupted Pathways	Functional Outcome
Primordial → Primary	Pre-GCs, oocyte	*Pten*, *Foxo3a*, *Kitl*, *Gdf9*, *Bmp15*	↑ Nuclear *FOXO3a*, ↓ *Kitl/Gdf9* expression	PI3K/AKT/FOXO3a	Inhibited activation, follicle dormancy or apoptosis

This table presents a phase-specific summary of the molecular disturbances induced by PFASs during folliculogenesis. Each developmental stage is associated with the impacted cell types, modified genes or proteins, interrupted signalling pathways, apparent functional effects, and corroborating references from animal or human model research.

**Table 3:** PFAS-induced molecular disruptions and associated clinical outcomes in female reproductive health.

PFAS-Induced Molecular Effect	Affected Structure/Axis	Clinical Consequence
Suppression of GnRH and LH/FSH secretion	Hypothalamus and pituitary	Delayed puberty, amenorrhea
Inhibition of FSHR–cAMP–CREB signalling	Granulosa cells	Poor response to stimulation, reduced E2 production
PPARγ activation	Ovarian tissue	Hypoaromatisation, hyperandrogenism
Disruption of GDF9/BMP15 and Cx43 signalling	Oocyte–cumulus complex	Poor oocyte quality, impaired maturation
Methylation of Amhr2/Esr1 promoters	Endometrium	Reduced receptivity, implantation failure
Elevated TNFα/IL6/ROS	Ovary/endometrium	Inflammation, oxidative stress, accelerated aging
Disruption of maternal miRNAs	Oocyte/extracellular vesicles	Transgenerational fertility effects

This table shows the most important changes to molecules that per- and PFASs cause at different levels of the female reproductive system and the clinical effects that go along with them. Disruptions affect neuroendocrine signalling (GnRH, FSH/LH), the production of steroids by granulosa and theca cells, communication between cumulus and oocyte, the receptivity of the endometrium, and epigenetic programming. These pathways are linked to a wide range of reproductive problems, such as not ovulating, having low oocyte quality, failing to implant, and having consequences on fertility that last for generations.
